# Parallax-Tolerant Weakly-Supervised Pixel-Wise Deep Color Correction for Image Stitching of Pinhole Camera Arrays

**DOI:** 10.3390/s25030732

**Published:** 2025-01-25

**Authors:** Yanzheng Zhang, Kun Gao, Zhijia Yang, Chenrui Li, Mingfeng Cai, Yuexin Tian, Haobo Cheng, Zhenyu Zhu

**Affiliations:** 1 Key Laboratory of Photoelectronic Imaging Technology and System, Ministry of Education of China, Beijing Institute of Technology, Beijing 100081, China; 3120220614@bit.edu.cn (Y.Z.); 3120225323@bit.edu.cn (Z.Y.); 3120220553@bit.edu.cn (C.L.); 3120220534@bit.edu.cn (M.C.); chb@bit.edu.cn (H.C.); 2 School of Innovation and Entrepreneurship, Southern University of Science and Technology, Shenzhen 518055, China; tianyx@mail.sustech.edu.cn; 3 Key Laboratory of Metallurgical Equipment and Control Technology, Ministry of Education, Wuhan University of Science and Technology, Wuhan 430081, China

**Keywords:** artifact elimination, color correction, image stitching, parallax, pinhole camera arrays

## Abstract

Camera arrays typically use image-stitching algorithms to generate wide field-of-view panoramas, but parallax and color differences caused by varying viewing angles often result in noticeable artifacts in the stitching result. However, existing solutions can only address specific color difference issues and are ineffective for pinhole images with parallax. To overcome these limitations, we propose a parallax-tolerant weakly supervised pixel-wise deep color correction framework for the image stitching of pinhole camera arrays. The total framework consists of two stages. In the first stage, based on the differences between high-dimensional feature vectors extracted by a convolutional module, a parallax-tolerant color correction network with dynamic loss weights is utilized to adaptively compensate for color differences in overlapping regions. In the second stage, we introduce a gradient-based Markov Random Field inference strategy for correction coefficients of non-overlapping regions to harmonize non-overlapping regions with overlapping regions. Additionally, we innovatively propose an evaluation metric called Color Differences Across the Seam to quantitatively measure the naturalness of transitions across the composition seam. Comparative experiments conducted on popular datasets and authentic images demonstrate that our approach outperforms existing solutions in both qualitative and quantitative evaluations, effectively eliminating visible artifacts and producing natural-looking composite images.

## 1. Introduction

**Figure 1:**
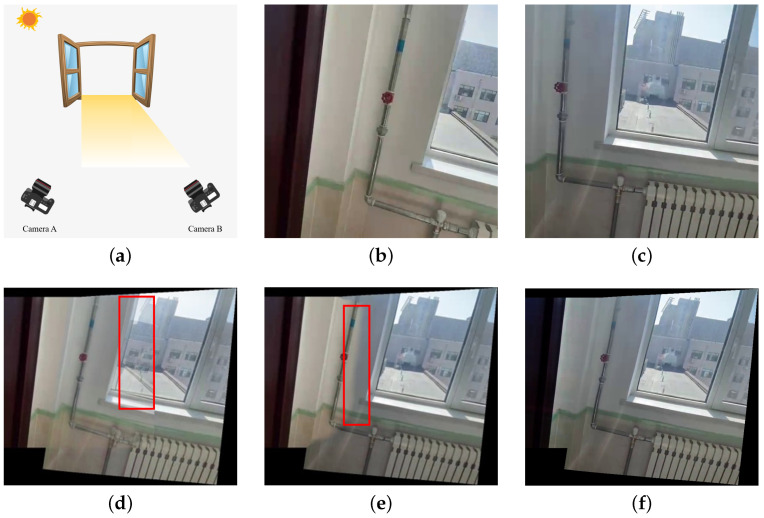
(**a**) An example of what causes parallax and color differences in images. (**b**) The target image with warm color tone. (**c**) The reference image with cool color tone. (**d**) The fusion result of the images using weighted averaging, with noticeable artifacts on the buildings outside the window shown in the red frame. (**e**) Composition result of UDIS++ [9], with noticeable color differences on the wall shown in the red frame. (**f**) Result of our parallax-tolerant color correction method.

Camera arrays are capable of simultaneously capturing views from multiple perspectives, rendering them indispensable in applications such as three-dimensional reconstruction [1], robotic vision [2], and target localization [3]. Depending on the type of camera, camera arrays can be classified into pinhole camera arrays and fisheye camera arrays [4]. Aiming at registering, aligning, and blending images from different perspectives into a wide field of view (FoV) and natural-looking panorama [5], image-stitching algorithms play a crucial role in the applications of camera arrays. Registration refers to the process of identifying corresponding similar regions between input image pairs. Alignment warps the input images such that the similar regions obtained through registration are maximally overlapped. Blending is tasked with the operation of merging the aligned images into a seamless stitching result, which is also the primary focus of this paper’s research. Since the images to be stitched are usually taken from different perspectives, the occlusion relationships and illumination conditions between them may vary significantly [6], causing parallax and color differences, respectively, both of which can result in noticeable seams in the stitching results. Figure 1a illustrates an example where camera A is placed in the shaded area, while camera B is placed in direct sunlight. The variations in the imaging optical paths and lighting conditions will, respectively, result in parallax and color differences between the photos they captured, as shown in Figure 1b,c. Images with parallax cannot achieve complete alignment so that the fusion results of the traditional weighted averaging method will have visible artifacts, as shown in Figure 1d. For images that contain parallax, blending is often performed using seam cutting methods. By  calculating the optimal seam, seam cutting methods divide the whole scene into separate parts, which effectively eliminates the parallax artifacts. Except for traditional seam calculating approaches based on dynamic programming (DP) [7] and GraphCut (GC) [8], deep seam prediction networks [9,10] have been proposed recently. However, for images with significant color differences, the seam will be clearly visible in the composition result, as shown in Figure 1e. Therefore, it is really necessary to implement color correction in the process of image blending.

During color correction for image stitching, one of the images to be stitched is chosen as the reference image, and the color style of the other image (target image) is meant to be corrected based on it. But, in terms of images with parallax, pixels at the same position in two images may represent different items. Consequently, it is irrational to simply convert the pixel values of the target image to be exactly the same as those of the reference image. Currently, color correction algorithms for image stitching can be classified into two categories: mean-based methods and feature-based methods. Mean-based methods [11,12,13,14] calculate color compensation parameters based on the differences between mean values of overlapping regions. In other words, the same set of correction coefficients are applied to all pixels. Therefore, they can only achieve satisfactory correction effects for images with a relatively uniform color difference distribution and cannot perform specific corrections for particular regions. Feature-based methods, on the other hand, first extract the features from either the distribution of the data [15,16,17,18] or the geometric structure of the images [19,20,21,22], and then fit a color mapping function based on the color differences between the extracted features. Feature-based methods can achieve specific color correction for particular regions, but the efficacy of correction is critically dependent on the quantity and quality of the extracted features. In addition, several deep learning-based color correction methods for image stitching have been proposed. Song et al. [23] proposed a weakly supervised color consistency correction framework for fisheye image stitching, which contains a pre-color correction stage for overlapping regions and a post-color correction stage for non-overlapping regions. Zheng et al. [24] proposed a panoramic algorithm integrating physics-driven and data-driven approaches for differently exposed panoramic low dynamic range (LDR) images. But the images to be stitched were assumed to be geometrically aligned in their work, which means they did not take the impact of parallax into consideration. Therefore, the deep learning method of pixel-wise color correction for pinhole images with parallax remains unexplored.

To overcome the limitations of traditional solutions and fill the gap of deep solutions, we propose a parallax-tolerant deep color correction framework abbreviated as PTDCC that comprises two stages. In the first stage, we propose a parallax-tolerant color correction network for overlapping regions. To achieve pixel-level color correction, we utilize a series of convolution layers and nonlinear activation layers as the backbone network to perform pixel-wise feature extraction on both images. Then, we construct a U-correction module [25] that takes the target image, reference image, and the root mean square error (RMSE) of their feature vectors as inputs. The network will output the gamma correction coefficients for each pixel within the overlapping regions. Additionally, we dynamically adjust the weight of the color difference constraint term in the overall loss function based on the RMSE of the feature vectors for each pixel. Specifically, for pixels with similar features, the weight of the color difference constraint term is increased, making the color correction result of the target image approach the reference image. Conversely, for pixels with dissimilar features, the weight of the color difference constraint term is decreased; therefore, the weight of the mutation constraint term is relatively increased, making the gamma correction coefficients of these pixels similar to those of adjacent pixels, preventing distortions in the result. Building on the loss function described above, our network can achieve pixel-level color correction while avoiding misalignment caused by parallax. In the second stage, based on the Markov Random Field (MRF) theory [26], we infer the gamma correction coefficients of the pixels in non-overlapping regions according to their neighborhoods. Specifically, we iteratively minimize the gradient similarity energy function which aligns the gradients of the boundary pixels between assigned and unassigned regions. By iteratively assigning gamma coefficients to the edge of unassigned regions layer by layer, we can eventually infer the gamma coefficients of the entire non-overlapping region, ensuring a harmonious and consistent correction for the target image. Thereby, we can realize color correction in the pixel level and obtain a seamless composition result, as illustrated in Figure 1f.

Additionally, we conduct extensive comparative experiments on the color-corrected target images and the composition outcomes, demonstrating our superiority to other solutions. Due to the limitations of existing evaluation metrics in measuring the visibility of stitching artifacts, we also designed a specific evaluation metric called Color Differences Across the Seam (CDCS) to measure the uniformity of transitions across the seam of composition results. The contributions of this paper are summarized as follows:

- We propose a parallax-tolerant weakly supervised pixel-wise color correction framework for overlapping regions. By distinguishing pixels based on the differences between their high-dimensional feature vectors, we can identify the presence of parallax, thereby achieving parallax-tolerant color correction for overlapping regions in the pixel level.
- We propose a gradient-based MRF approach to iteratively infer gamma correction coefficients of non-overlapping regions layer by layer. By minimizing the gradient similarity energy function, we can ensure a smooth integration of colors throughout the entire target image, thereby achieving a natural-looking panorama.
- We design a specific evaluation metric to measure the uniformity of transitions across the seam of composition results, thereby converting the subjective evaluation of the visibility of stitching artifacts, as perceived by the human eyes, into an objective metric that can be represented quantitatively.

## 2. Related Works

### 2.1. Traditional Color Correction Method

#### 2.1.1. Mean-Based Methods

Global luminance compensation (GLC) [12] transforms the images to be stitched into the HSV color space and computes the average V-component of overlapping regions, denoted as $v_{1}$ and $v_{2}$, to obtain a global gamma correction coefficient $\log_{v_{1}}v_{2}$. This method only addresses brightness differences and thus fails to effectively correct images with significant color tone variations [21]. Shen et al. [11] propose a color correction algorithm based on the difference in gray values in overlapping regions, and  compensate the target image in RGB channels with it. Furthermore, global color compensation (GCC) [13] addresses the problem of uneven luminance and color in stitched images by compensating color differences in the Lab color space. The average luminance difference $L_{avg}$ along with average color differences $A_{avg}$ and $B_{avg}$ are calculated and added to the image with lower brightness. Similarly to GCC, Lin et al. [14] implement compensation based on the average Lab differences in regions near the composition seam.

In mean-based methods, the correction coefficients are derived from the average value of all pixels in overlapping regions, which could result in unsatisfactory compensation or even distortion if the color differences are unevenly distributed.

#### 2.1.2. Feature-Based Methods

**Distribution-based Methods.** Histograms are often used to describe the distribution features of an image. For example, the hybrid histogram matching (HHM) algorithm [15] first utilizes cumulative histograms of overlapping regions to derive a global mapping function, then remove the staircase-related noisy mapping pairs for local color straightening. Yang et al. [16] employ histogram specification to equalize the color distribution in overlapping regions and balance the color of the entire stitched image by constructing a distribution function composed of a linear function and a gamma function. Besides the aforementioned histogram-based methods, Jeong et al. [17] oversegment the target image and reference image into superpixels, and utilize the Coherent Point Drift (CPD) [27] algorithm to deform the color distribution of the target image to the reference image. Yu et al. [18] present an algorithm based on the Wallis filter which utilizes the mean and standard deviation of the images to maintain consistent color across multiple hyperspectral remote sensing images.

Distribution-based methods estimate the mapping functions by analyzing the distribution of different color pixels within overlapping regions. These approaches necessitate that the scenes in overlapping areas of the images be highly consistent. Consequently, image pairs with substantial parallax may cause inaccurate mappings.

**Geometry-based Methods.** SIFT [28] is the most commonly used feature extractor in geometry-based methods. In [19], the triangulation of the feature points is employed to segment the color regions of the images. After removing the areas with large differences in shape, the transformation model is calculated based on the matched color pairs. Similarly, Liu et al. [20] segment the target image into several regions based on the SIFT features and compensate for the color differences by adding the mean difference of the feature points to the corresponding regions. Wang et al. [21] employ a vector field consensus algorithm [29] to remove the mismatches of SIFT feature points and a quartic color mapping model is fitted based on the remaining feature points. Lo et al. [22] propose a photometric compensation method that combines intensity modeling and color transfer. The process involves two key steps: first, modeling intensity with spherical harmonic functions to minimize intensity differences; second, applying color transfer to reduce color differences.

Nevertheless, traditional feature extraction algorithms relying on intricate geometric features struggle to adapt to scenes lacking sufficient geometric structures, as well as other natural images that have low texture, poor lighting, or low resolution [9].

### 2.2. Deep Color Correction Method

Inspired by Zero-DCE [30], a weakly supervised color correction framework [23] is proposed by Song et al. In contrast to pinhole image stitching, their dataset comprises fisheye images captured from six distinct angles, each separated by 60°. As a result, while three of these images serve as inputs for the stitching network, the remaining three are utilized as weak supervisions to harmonize the stitched images with each other. In [24], they propose a physics-driven deep panoramic imaging framework for low dynamic range scenes, in which intensity mapping functions [31] are estimated as the physics-driven preprocessing and a multiscale exposedness-aware network is utilized to perform data-driven refinement. However, they assume the low dynamic range images to be stitched are geometrically aligned. Therefore, neither of these two methods can deal with the color correction situation of pinhole images with parallax.

## 3. Methods

**Figure 2:**
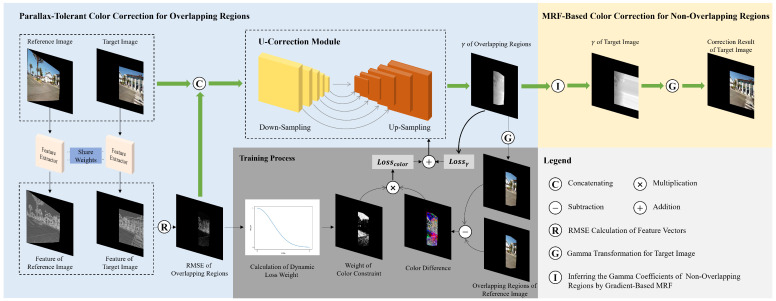
Overview of the proposed method.

Since the primary focus of this paper is color correction during the image blending stage, we employ the state-of-the-art parallax-tolerant method, UDIS++ [9], to perform image registration and alignment. Subsequently, color correction and blending are carried out using our proposed method. The pipeline of our proposed framework is shown in Figure 2, which is divided into two stages: parallax-tolerant color correction for overlapping regions and MRF-based color correction for non-overlapping regions. The inputs for the first stage include the target image $I_{t}$ and the reference image $I_{r}$. Subsequently, both $I_{t}$ and $I_{r}$ pass through the same feature extractor to obtain their respective pixel-level features. The RMSE of the feature vectors in overlapping regions, concatenated with $I_{t}$ and $I_{r}$, are taken as the input to the U-correction module, where the gamma correction coefficients of overlapping regions $\gamma_{o}$ are regressed as the outputs. During the training process of the U-correction module, a loss function $Loss_{color}$ constrains the pixels with smaller RMSE in overlapping regions, ensuring that their correction results are similar to those of the reference image. Additionally, another loss function $Loss_{\gamma }$ constrains all pixels in the target image to prevent mutation outliers in the correction results. In the second stage, correction coefficients of non-overlapping regions are inferred by a gradient-based MRF approach, outputting the gamma correction coefficients of the entire target image $\gamma_{t}$. The color correction result $I_{c}$ is represented by  

(1) $$I_{c}={I_{t}}^{\gamma_{t}}.$$

### 3.1. Parallax-Tolerant Color Correction for Overlapping Regions

#### 3.1.1. Motivation

Since the parallax implies that the pixels in overlapping regions of the input images represent different objects, the feature differences between pixels with parallax should be significant. For example, in Figure 1c, the object on the far left outside the window is a tower, whereas, in Figure 1b, the tower is blocked due to the change in the shooting angle. Accordingly, we utilize a convolutional architecture to calculate the feature vectors for each pixel, and the RMSE of the feature vectors at the same position is employed to determine whether there is parallax. For pixels with similar feature vectors, the RGB values of the reference image are taken as the ground truth for the color correction result of the target image. For pixels with significant differences between feature vectors, the reference image is no longer taken as the ground truth. Instead, considering the similarity of illumination conditions between adjacent pixels, the gamma coefficients of adjacent pixels are taken as references. In other words, the reference image is taken as weak supervision for the color correction result.

#### 3.1.2. Network Architecture

**Figure 3:**
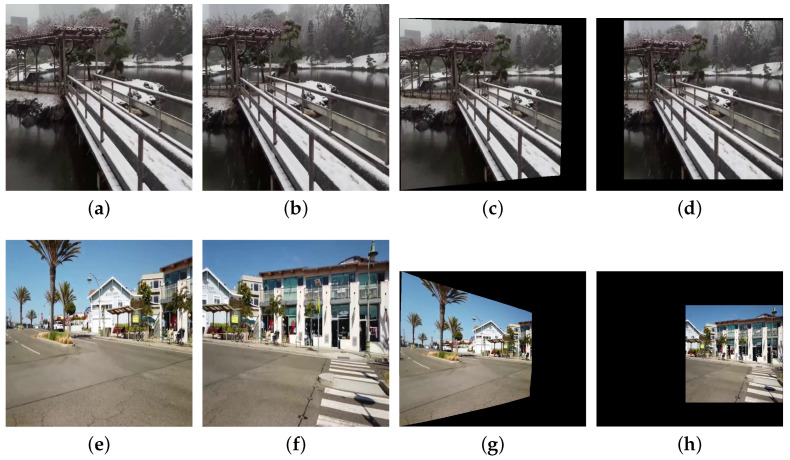
A comparison example of small baseline scenes and large baseline scenes. (**a**,**b**) represent a pair of target and reference images under a small baseline scene, both with a resolution of 512 × 512. (**c**,**d**) are the corresponding alignment results. (**e**,**f**) represent a pair of images under a large baseline scene, and (**g**,**h**) are the corresponding alignment results. Effective regions containing the scene information in both (**d**,**h**) have dimensions of 512 × 512.

Since we use UDIS++ [9] for image registration and alignment, it is necessary to clarify the relationship between the alignment results and the original images. Figure 3 illustrates the differences in alignment results between two types of original images. The sizes of original images (Figure 3a,b,e,f) are all 512 × 512. However, the viewpoint distance between Figure 3a,b is relatively short, with a large overlapping region, referred to as a small baseline scene [32]. In contrast, the viewpoint distance between Figure 3e,f is much larger, with a smaller overlapping region, referred to as a large baseline scene. Accordingly, the alignment results for Figure 3a,b are smaller in size, with dimensions of 599 × 554 (as shown in Figure 3c,d), while the alignment results for Figure 3e,f are larger, with dimensions of 979 × 815 (as shown in Figure 3g,h). Therefore, the input size of our color correction model varies depending on the length of the baseline, and the sizes of each input pair are not identical. This requires our model to handle inputs of arbitrary sizes and output color correction results of the same size in the meantime.

**Figure 4:**
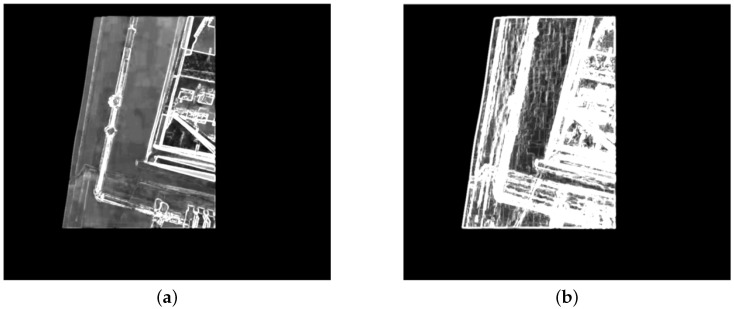
The RMSE distributions in overlapping regions of Figure 1b,c utilizing feature extraction layers at different depths. (**a**) The RMSE distribution obtained using a shallow feature extractor. (**b**) The RMSE distribution obtained using a deep feature extractor.

In order to meet the above requirements, we first extract pixel-wise features using a series of convolution layers and nonlinear activation layers as the feature extractor shown in Figure 2. To be more specific, we employ four pairs of convolution layer and ReLU layer [33] to compute high-dimensional features for each pixel. Since it is essential for the feature extractor to handle inputs of arbitrary sizes, we avoid introducing linear layers, which typically require fixed input dimensions. Additionally, because the output feature map is required to maintain the same width and height as the input image, ensuring that each pixel has a corresponding feature vector for subsequent loss function calculation, we refrain from using pooling layers, which would reduce the spatial dimensions of the feature map. Figure 4a illustrates the RMSE distribution of feature vectors obtained from the first two pairs of convolution layer and ReLU layer. Due to the extraction of relatively shallow texture features, it can only mark small portions of edge parallax. In contrast, Figure 4b presents the distribution map derived from the complete feature extractor. By performing deeper feature extraction, we can capture features beyond just the edges, enabling precise identification of parallax across the entire overlapping region.

Subsequently, the RMSE of the feature vectors along with $I_{t}$ and $I_{r}$ are concatenated as the input of our correction coefficient prediction network, which is a U-correction module including five down-sampling and up-sampling blocks. Unlike traditional u-net [25], we not only perform down-sampling through max-pooling layers but also introduce a dilation that increases with the sampling depth, further expanding the receptive field of the network. Correspondingly, we introduce a decreasing dilation in the up-sampling block, allowing the dimensions of different feature maps to align through skip connections. Additionally, since the input to the network is the combination of $I_{t}$, $I_{r}$, and the RMSE of their feature vectors, and the output is the gamma correction coefficient for each pixel in overlapping regions of the target image across RGB channels, we modify the model’s input channel to 7 and the output channel to 3. The parameter settings of the convolutional layers in each block are shown in Table 1. The convolutional kernel size of the final block, up_sampling block 0, is 1 × 1, serving solely for dimensionality reduction. The max-pooling layers, dilated convolution layers, and skip connection technique in the module effectively increase the reception field of the network, making it well suited to the large-scale color correction requirements of this study and, in the meantime, realizing pixel-wise color correction. In addition, since the sizes of the input image pairs may be different, the batch size for input data should be set to 1. Consequently, we remove the batch-normalization layers from the original u-net to avoid instability in mean and variance estimation caused by single batch size.

#### 3.1.3. Loss Functions

The loss function of our weakly supervised color correction network consists of two parts: the color difference constraint term and the mutation constraint term, expressed by the following equation:

(2) $$Loss_{overlap}=\alpha Loss_{color}+\beta Loss_{\gamma }.$$

(a) Color Constraint with Dynamic Weights

**Figure 5:**
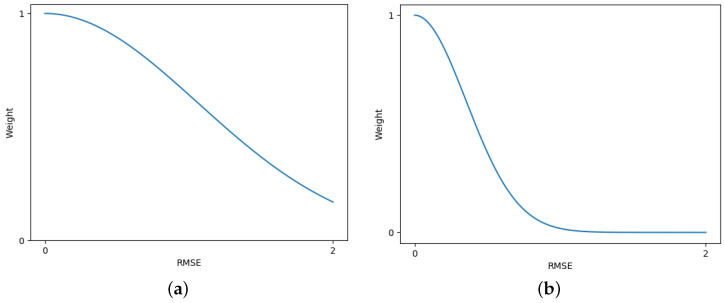
The effect of the parameter σ on the weight of the color difference loss. (**a**) σ=1.5. (**b**) σ=0.5.

First, to avoid directly correcting the color of regions with parallax, which would cause artifacts, we designed a parallax-tolerant color correction loss function with dynamically changing weights:

(3) $$RMSE=\sqrt{\frac{\sum_{k=1}^{N}{\left(F_{t,k}-F_{r,k}\right)}^{2}}{N}},$$

(4) $$w=e^{-{\left(\frac{RMSE}{\sigma }\right)}^{2}},$$

(5) $$\begin{array}{cc} \; & Loss_{color}=\sum_{i,j}w(i,j)\cdot \left(\right|R_{c}(i,j)-R_{r}(i,j)|+|G_{c}(i,j)-G_{r}(i,j)|+|B_{c}(i,j)-B_{r}(i,j)\left|\right), \end{array}$$

where $F_{t,k}$ and $F_{r,k}$ represent the k−th channel feature vectors of the target image and the reference image, *N* is the total number of channels, and RMSE is the root mean square error between the feature vectors of each pixel. *w* represents the weight of the color difference loss for each pixel in the total loss function, decreasing with increasing RMSE, and σ is the influence factor controlling the weight distribution. $R_{c},G_{c},B_{c}$ and $R_{r},G_{r},B_{r}$ represent the RGB values of the color correction result and the reference image, respectively. Figure 5 shows how the mapping between RMSE and *w* changes with different values of σ. When σ is large, the model has a higher tolerance for feature differences, resulting in a generally larger weight for $Loss_{color}$, which may cause artifacts due to taking the reference image as the ground truth of the parallax regions. In contrast, when σ is small, the model has a lower tolerance for feature differences, potentially leading to insufficient color correction in small parallax regions. Therefore, an appropriate σ value needs to be chosen so that small parallax regions take the reference image as the ground truth, while large parallax regions do the opposite.

Therefore, the above loss function defines the weight of the color difference loss for each pixel in the overall loss function based on the RMSE of the feature vectors. The smaller the parallax or the more similar the feature vectors, the greater the weight of the color difference loss, making the color correction result at the corresponding position of the target image closer to the reference image.

(b) Mutation Constraint

Furthermore, besides constraining pixels with similar feature vectors through the parallax-tolerant color correction loss function, there is also a need to impose constraints on pixels with significant feature differences, in order to avoid the occurrence of outliers for pixels with large feature disparities. Considering that, in real-world scenarios, the illumination conditions of adjacent pixels should be analogous, we propose the following mutation constraint for γ:

(6) $$Loss_{\gamma }=\sum_{i,j}\left(\right|\gamma_{i,j}-\gamma_{i,j-1}\left|+\right|\gamma_{i,j}-\gamma_{i-1,j}\left|\right),$$

where i,j represent the row and column coordinates of the pixel, and $\gamma_{i,j}$ represents the γ value at (i,j).

This loss function encourages the correction coefficients of adjacent pixels to be approximately similar, thereby preventing the occurrence of extreme correction coefficients and color mutation in the corrected results. Since all pixels are required to adhere to this constraint, we do not introduce dynamic weights that could potentially become minimal into this loss function, but instead apply the same weight to all pixels.

### 3.2. MRF-Based Color Correction for Non-Overlapping Regions

**Figure 6:**
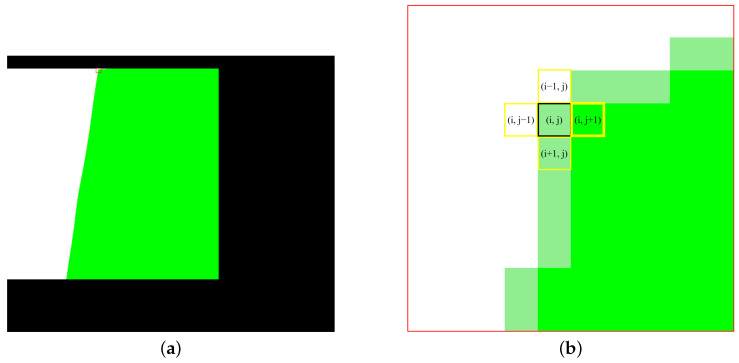
An illustration of overlapping regions and non-overlapping regions. (**a**) Overview image, with a red box on the upper side of the junction between the two regions. (**b**) The detailed enlarged view of the red box in (**a**).

In this study, we adopt a gradient-based MRF approach to iteratively assign gamma coefficients to non-overlapping regions layer by layer. Taking Figure 1b as an example, the mask of overlapping and non-overlapping regions is illustrated in Figure 6a. The green area represents overlapping regions, which is also the area that has already been assigned gamma coefficients. The white area represents non-overlapping regions, which is also the area to be inferred. The light green area represents the nearest pixels in non-overlapping regions adjacent to the overlapping regions, which are also the pixels that need to be assigned coefficients first. The fundamental assumption of MRF is that each pixel’s value depends only on its neighboring pixels, and is independent of all other pixels [26]. This local dependency allows us to infer the gamma coefficients of unassigned regions by starting from the boundary between assigned and unassigned regions and iterating layer by layer. The enlarged view of the red boxed area in Figure 6a is shown in Figure 6b, which is used to demonstrate the inference process of the correction coefficients in non-overlapping regions. Taking the light green pixel marked by the black box in Figure 6b as an example, its 4 neighborhoods are highlighted by yellow boxes, among which the adjacent pixel on the right has already been assigned coefficients (indicated by the bold yellow box). Therefore, this pixel will be assigned gamma coefficients based on its right-side neighboring pixel.

First, we initialize the process at boundary conditions. The boundary pixels at the interface between the overlapping and non-overlapping regions are assigned gamma coefficients based on their adjacent assigned pixels. We assume that the boundary pixels in the unassigned regions exhibit smoothness and consistency in gradient with their neighboring assigned pixels. This gradient similarity is represented by an energy function E(γ), which reflects the interaction between neighboring pixels. Specifically, we use a squared gradient difference form for the energy function:

(7) $$E\left(\gamma \right)=\sum_{j\in N(i)}|\nabla_{i,j}{\left(\gamma \right)|}^{2}=\sum_{j\in N(i)}{\left|\gamma_{i}-\gamma_{j}\right|}^{2}$$

where N(i) represents the neighborhoods of pixel *i*, typically its four adjacent pixels (above, below, left, and right), and $\nabla_{i,j}\left(\gamma \right)$ is the gradient difference between pixel *i* and *j*. Based on this energy function, the boundary pixels in unassigned regions are assigned gamma coefficients by minimizing the gradient difference with respect to their adjacent assigned pixels:

(8) $$\gamma_{i}=\arg\min_{\gamma }\sum_{j\in N(i)}|\nabla_{i,j}{\left(\gamma \right)|}^{2}=\arg\min_{\gamma }\sum_{j\in N(i)}{\left|\gamma -\gamma_{j}\right|}^{2}$$

Once the boundary pixels are initialized, we proceed to the next step of the iterative process. In each subsequent iteration, the remaining unassigned pixels are inferred by minimizing the same energy function (8) over their neighborhoods, which include the known values in assigned regions and the previously assigned values in unassigned regions. This process iterates layer by layer, starting from the unassigned boundary closest to the assigned regions and gradually filling the entire non-overlapping region until all pixels are assigned. To ensure stability and convergence of the inference process, we adopt a synchronous update strategy, where all pixels in each boundary layer of unassigned regions are updated simultaneously in each iteration.

Through this gradient-based MRF layer-by-layer iterative process, we can infer reasonable gamma coefficients for the entire target image, enabling us to perform color correction as described in (1). A concise description of the procedure can be found in Algorithm 1.
**Algorithm 1:** Inference of Gamma Coefficients by Gradient-Based MRF 
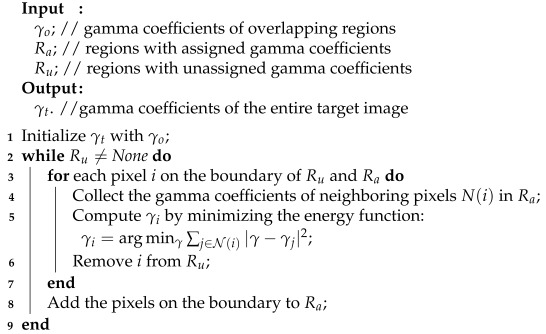


## 4. Experimental Results

### 4.1. Dataset and Implementation Details

*Dataset:* Since the research object in this study is real-world stitching scenarios with parallax and color differences, we utilize UDIS-D [34] as the benchmark dataset and employ UDIS++ [9] to compute the warped results as well as the composition masks for each image pair. With the objective of optimizing our network’s capacity to discern nuances in color feature representation, we meticulously crop the dataset into 3019 training image pairs and 32 test image pairs that exhibit significant color differences.

*Details:* Our parallax-tolerant color correction network is trained for 50 epochs using the Adam [35] optimizer with an exponentially decaying learning rate that starts at $10^{-4}$. All implementations are performed with PyTorch 1.13.1+cu117 on a single NVIDIA GeForce RTX 2080 Ti GPU. The average time for training one epoch is 12 min. To balance the influence of $Loss_{color}$ and $Loss_{\gamma }$ on the correction results, we conduct a series of experiments with controlled variables on the loss function weights. Ultimately, the influence factor σ is set to 0.85, while the hyper-parameters α and β are, respectively, set to 50 and 10.

### 4.2. Comparative Experiments

To evaluate the performance of the proposed method, we qualitatively and quantitatively compare our color correction results with the uncorrected original UDIS++ [9], and mean-based methods GLC [12] and GCC [13], as well as feature-based method HHM [15]. In addition, we incorporate the Transformer-based model TransUNet [36] into our correction framework, which not only validates the effectiveness of our correction framework but also demonstrates that our proposed CNN-based PTDCC is more suitable than Transformer-based methods for addressing the color correction problem in image stitching. Considering that the input image size in our framework is arbitrary, while the linear layers in Transformer-based methods require a fixed input size, and the Transformer module in TransUNet is pre-trained on the ImageNet [37], we resize the input images to the standard ImageNet size of 224 × 224 so that the U-correction module in our correction framework can be replaced with TransUNet. Afterward, the gamma correction coefficients output by the network are resized back to the original image size through interpolation. To ensure the rigor of the comparison, other parts of the framework and implementation details remain consistent with our PTDCC.

#### 4.2.1. Qualitative Comparison

To demonstrate the effectiveness of our color correction network, we categorize the test images into three groups: images with evenly distributed brightness differences, images with unevenly distributed brightness differences, and images with significant hue differences, whose correction difficulties range from low to high.

**Figure 7:**
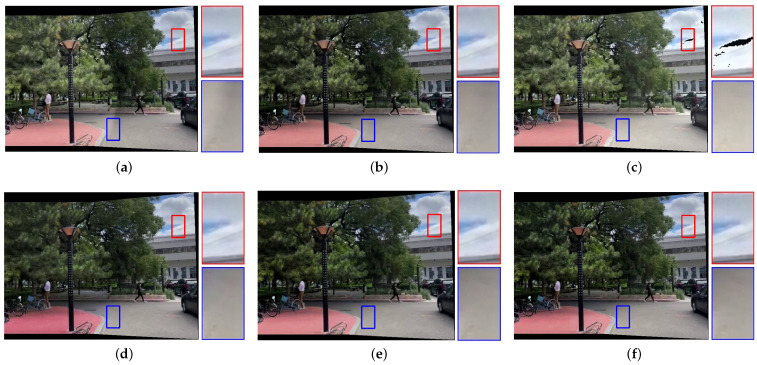
Qualitative comparison results of images with evenly distributed brightness differences. (**a**) UDIS++. (**b**) GLC. (**c**) GCC. (**d**) HHM. (**e**) TransUNet. (**f**) Ours.

**Figure 8:**
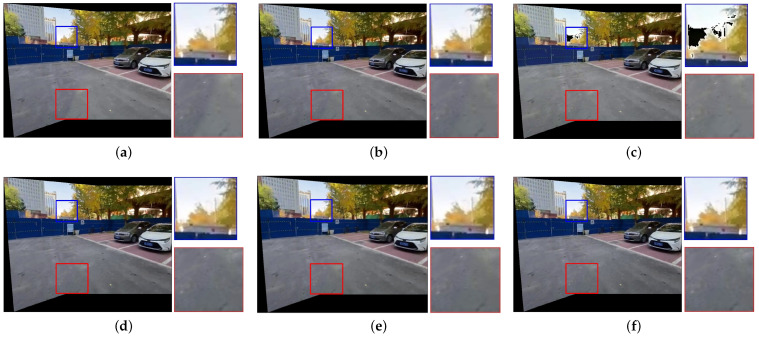
Qualitative comparison results of images with unevenly distributed brightness differences. (**a**) UDIS++. (**b**) GLC. (**c**) GCC. (**d**) HHM. (**e**) TransUNet. (**f**) Ours.

**Figure 9:**
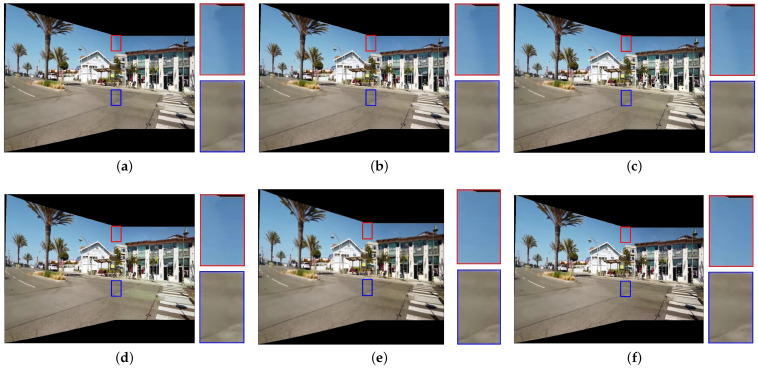
Qualitative comparison results of images with unevenly distributed brightness differences and large parallax. (**a**) UDIS++. (**b**) GLC. (**c**) GCC. (**d**) HHM. (**e**) TransUNet. (**f**) Ours.

**Figure 10:**
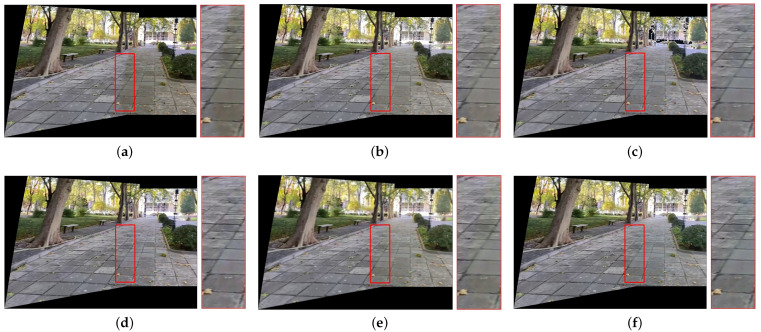
Qualitative comparison results of images with significant hue differences. (**a**) UDIS++. (**b**) GLC. (**c**) GCC. (**d**) HHM. (**e**) TransUNet. (**f**) Ours.

**Figure 11:**
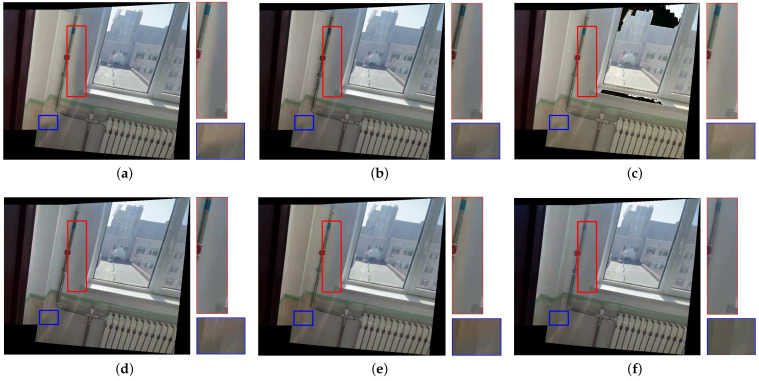
Qualitative comparison results of images with significant hue differences and large parallax. (**a**) UDIS++. (**b**) GLC. (**c**) GCC. (**d**) HHM. (**e**) TransUNet. (**f**) Ours.

Figure 7, Figure 8, Figure 9, Figure 10 and Figure 11 present the comparison of our results with other methods. As shown in Figure 8b, Figure 9b, Figure 10b and Figure 11b, the GLC algorithm, which solely adjusts the brightness channel based on the mean value, is inadequate in addressing unevenly distributed brightness differences and hue discrepancies, leading to conspicuous stitching artifacts. Although GCC performs color correction in the Lab color space, it calculates the correction coefficients based on the average differences of all pixels in overlapping regions, which can result in inadequate compensation and noticeable stitching artifacts as demonstrated in Figure 9c. In addition, since GCC performs color correction by adding the compensation parameters to the original image, areas close to white may have distortion after correction, as shown in Figure 7c, Figure 8c, Figure 10c and Figure 11c. The feature-based method HHM fits the mapping function across all three RGB channels, enabling it to effectively correct images with substantial color discrepancies and minimal parallax. However, for images with significant parallax, this method is prone to generating erroneous mappings, leading to conspicuous stitching artifacts in the composite results, as illustrated in Figure 9d and Figure 11d. As for TransUNet, it can achieve a certain degree of correction across various scenarios, which validates the effectiveness of the correction framework we proposed. However, the constraint of fixed input size may lead to the loss of feature information during the resizing process, which results in imperfections in its correction outcomes. For instance, visible color differences persist in the ground region of Figure 8e and Figure 10e, and in the wall region of Figure 11e. Conversely, our color correction network mitigates color differences across all three RGB channels through pixel-wise gamma correction and dynamically adjusts the weights of color mutation depending on the magnitude of the parallax without feature information loss; thus, we can effectively eliminate stitching traces and artifacts in the final outputs.

#### 4.2.2. Quantitative Comparison

In line with the qualitative comparison experiments, the quantitative comparison experiments also categorize the test data into three groups: easy group, moderate group, and hard group, based on the complexity of color correction, respectively.

(a) Existing Evaluation Metrics

As for the overlapping regions, since our color correction network takes the reference image as weak supervision, we employ PSNR and SSIM to evaluate the similarity of overlapping regions. The results, presented in Table 2, demonstrate that our color correction network delivers superior performance across various levels of color correction complexities.

Additionally, for the overall composition results, we utilize image naturalness evaluation metrics BRISQUE [38] and NIQE [39], which are often used to measure the similarity between composite images and natural images. The results, displayed in Table 3, indicate that our color correction method produces results that most closely resemble natural images, outperforming the other methods, which is consistent with our qualitative comparison findings.

(b) Innovative Metric

Although existing evaluation metrics can reflect the strengths and weaknesses of different algorithms to some extent, they still have limitations and are not fully applicable to image-stitching scenarios with parallax and color differences.

**Figure 12:**
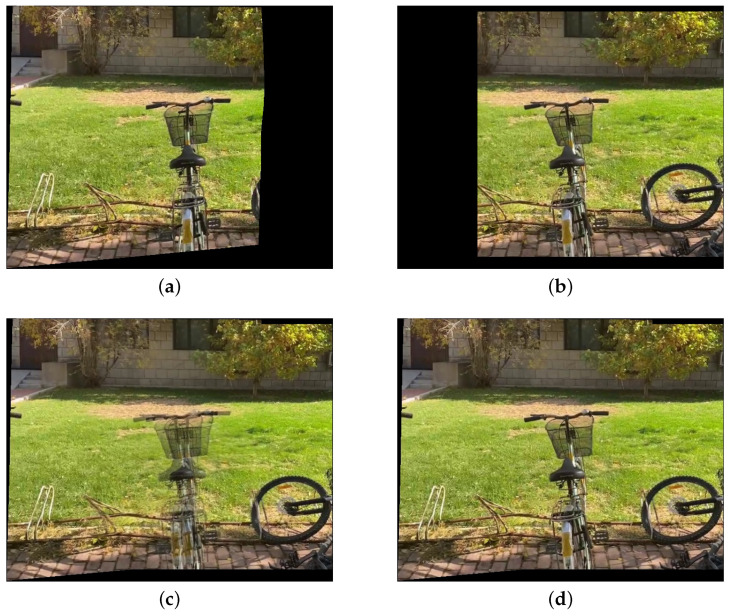
An example of images with noticeable parallax but no color difference. (**a**) Reference image. (**b**) Target image. (**c**) The fusion result obtained by the weighted average method. (**d**) Ours.

Figure 12 shows a pair of target and reference images. Although both images focus on the bicycle as the main subject, they are taken from different angles. As can be seen in Figure 12c, there is noticeable parallax but no color difference between the two images. Therefore, we can use the seam-based method to obtain a composition result without noticeable stitching artifacts, as shown in Figure 12d. However, for such images, the evaluation metrics PSNR and SSIM, with the reference image as the ground truth, yield poor results. The PSNR of Figure 12b is only 18.66, and the SSIM is only 0.608. These results contradict our original intention of measuring the visibility of stitching artifacts. Therefore, we believe that PSNR and SSIM are not entirely suitable for evaluating image pairs with parallax, as it is possible to obtain results with no stitching artifacts even when PSNR and SSIM are low.

**Figure 13:**
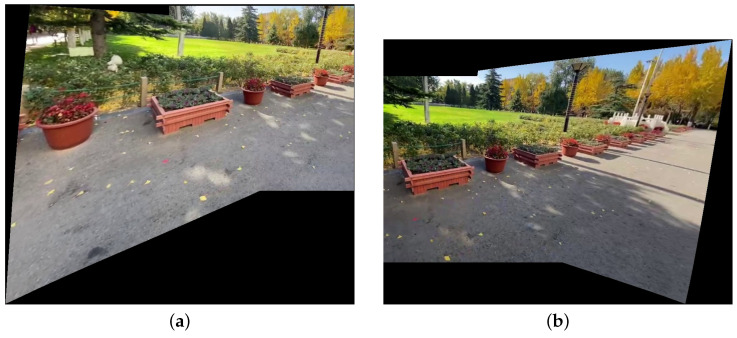
An example illustrating how contours of the effective region affect BRISQUE and NIQE. (**a**) BRISQUE = 31.57, NIQE = 3.00. (**b**) BRISQUE = 40.19, NIQE = 4.18.

As for BRISQUE and NIQE, although they can measure the naturalness of an image, the contours of conventional images in natural scenes are often standard rectangles. However, in image-stitching scenarios, image alignment inevitably causes image deformation, so the contour of the effective region (the area containing the scene) in the stitching result is no longer a standard rectangle, and it varies with the relative position of viewpoints. Figure 13 shows an example of two images with similar content but significantly different contours. In Figure 13a, the upper boundary, left boundary, and right boundary of the effective region closely match the outer contour, with only the bottom boundary being irregular. In Figure 13b, only the left boundary matches the outer contour, while the other three boundaries are irregular. Due to the differences in the contours, the BRISQUE and NIQE of Figure 13a are much smaller than those of Figure 13b. However, in this study, we aim to compare the visibility of stitching artifacts. Considering that both images display similar content and show no noticeable stitching artifacts, their evaluation results should be similar, which is inconsistent with the current outcomes. Therefore, we can conclude that BRISQUE and NIQE also have certain limitations.

To overcome the limitations of existing evaluation metrics, we innovatively propose an evaluation metric specifically used to measure the smoothness of the transitions across the composition seam, called Color Differences Across the Seam (CDCS). CDCS focuses solely on measuring the prominence of stitching artifacts, unaffected by parallax and contour shapes. It is defined as the ratio of the color differences across the seam to the length of the seam, which can be calculated as follows:

(9) $$CDCS=\frac{\sum_{i=0}^{L}RMSE_{i}}{L},$$

where *L* represents the length of the seam, and the color difference at the *i*-th row is determined by the RMSE of the average values of the five pixels closest to the seam on both sides. Since the UDIS-D dataset only includes the most common image-stitching scenarios, where the relative positions of the images to be stitched are approximately horizontal, the composition seams are curves that extend from the top to the bottom of the images. Therefore, here we calculate the color differences between the pixels on the left and right sides of the seam:

(10) $$RMSE_{i}=\sqrt{\frac{{\left(\bar{Left_{i}}-\bar{Right_{i}}\right)}^{2}}{3}}$$

where $\bar{Left_{i}}$ and $\bar{Right_{i}}$ represent the average RGB values of the five closest pixels to the seam on the left and right side, respectively (in cases where the images are positioned vertically relative to each other, the reference for calculating CDCS can be adjusted to the pixels on the upper and lower sides of the seam). Consequently, we can transform the subjective assessment of “the visibility of stitching artifact”, which is ordinarily evaluated by human perception, into an objective metric that can be quantitatively expressed in numerical terms, thereby facilitating more precise comparisons. The CDCS of Figure 12d is 10.32, while the CDCS of Figure 13a is 10.26 and that of Figure 13b is 10.13. The similar results for the three images with no noticeable stitching artifacts also confirm the scientific validity and effectiveness of CDCS.

The comparison results of the CDCS for the test set are shown in Table 4. Our color correction results consistently outperform the other methods, exhibiting the smallest color differences across the seam.

#### 4.2.3. Runtime Comparison

To evaluate the computational efficiency of different methods, we also compare the average time they spent on a single pair of input images. All comparison methods are executed on the same device equipped with an Intel Core i9-10900K CPU (Santa Clara, CA, USA) and an NVIDIA GeForce RTX 2080 Ti GPU (Santa Clara, CA, USA), with the results presented in Table 5. The results indicate that the two mean-based methods, GCC and GLC, exhibit the shortest processing times due to their lower algorithmic complexity. In contrast, the HHM method, which provides better correction performance, takes a longer processing time. Among the methods based on our correction framework, TransUNet has a higher computational cost, while our PTDCC, with fewer parameters than TransUNet, executes in less than half the time. After considering both correction accuracy and computational efficiency, we conclude that PTDCC remains the most optimal choice for correcting color differences between images with parallax.

### 4.3. Experiments on Authentic Images

**Figure 14:**
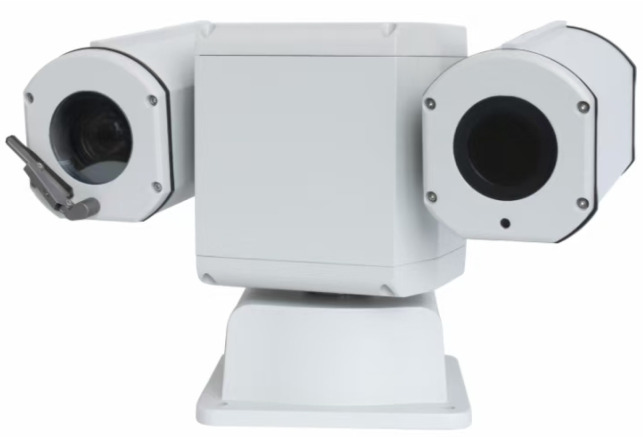
The stereo pinhole camera array we utilized.

In addition to conducting comparative experiments on the publicly available UDIS-D dataset, we also tested our color correction strategy using the stereo pinhole camera array shown in Figure 14. The camera array consists of two pinhole cameras placed parallel to each other at a certain distance, simulating the most common image-stitching application scenarios. Both cameras have identical lens parameters, including a focal length of 25 mm, an aperture value of 1.85, and an image resolution of 1920 × 1080.

**Figure 15:**
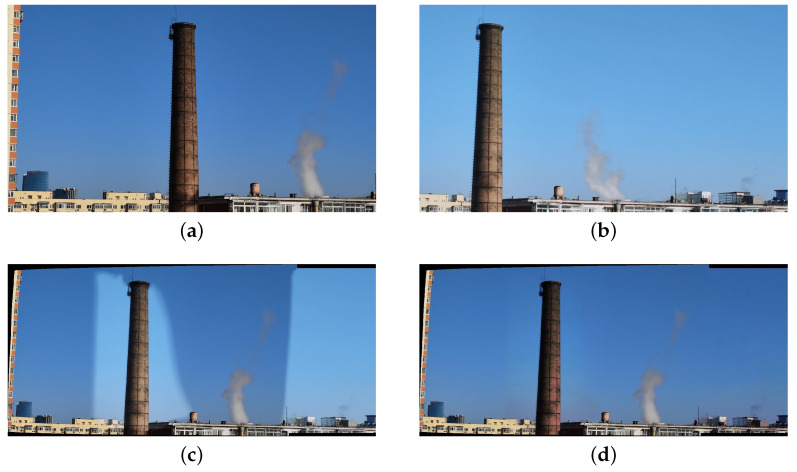
Images captured by our camera array and corresponding composition results. (**a**) Reference image. (**b**) Target image. (**c**) Composition result of UDIS++. (**d**) Ours.

Figure 15a,b illustrate a pair of images with obvious color differences captured by the camera array. Therefore, the result of UDIS++ (Figure 15c) exhibits relatively noticeable stitching seams. In contrast, the method we proposed effectively addresses color correction, yielding a seamless composition result, as shown in Figure 15d. Therefore, our method has been proven to be applicable to various pinhole imaging devices and shooting conditions.

### 4.4. Ablation Studies

**Figure 16:**
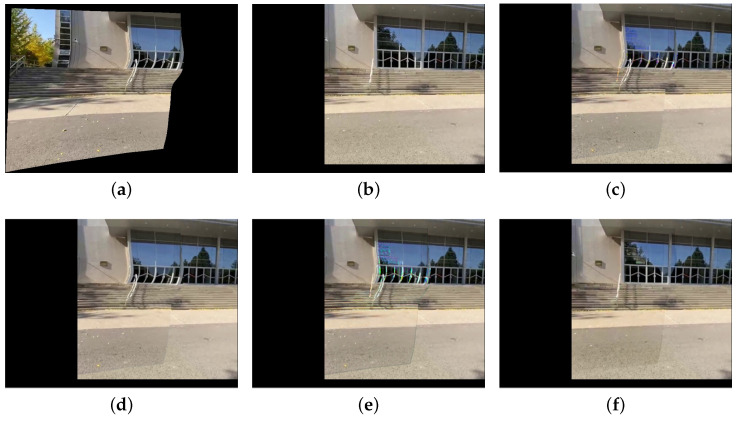
Ablation studies on parallax-tolerant color correction for overlapping regions. The color correction results for overlapping regions of the target image under different conditions are shown in the figure. (**a**) Reference image. (**b**) Target image. (**c**) w/o *w* and $Loss_{\gamma }$. (**d**) w/o *w*. (**e**) w/o $Loss_{\gamma }$. (**f**) Ours.

Firstly, we conduct ablation studies on the color constraint and mutation constraint by invalidating the dynamic weights *w* and mutation constraint $Loss_{\gamma }$, respectively. As shown in Figure 16, the correction result trained without dynamic weights and mutation constraint exhibits noticeable ghosting and slight distortion, while overlapping regions of the result obtained without dynamic weights is exactly the same as the reference image; thus, the image is noticeably uncoordinated. As for the result trained by only implementing dynamic weights, only the pixels without parallax are constrained, so that there are more noticeable distortions in regions with large parallax compared with the result trained without dynamic weights and mutation constraint. In contrast, our method not only corrects color differences but also eliminates ghosting and distortion, which means that the dynamic weights calculated by high-dimensional feature differences effectively eliminate the ghosting effects caused by indiscriminate correction while sudden changes and noise points in the image, as well as uneven transitions, are thoroughly suppressed by mutation constraint.

**Figure 17:**
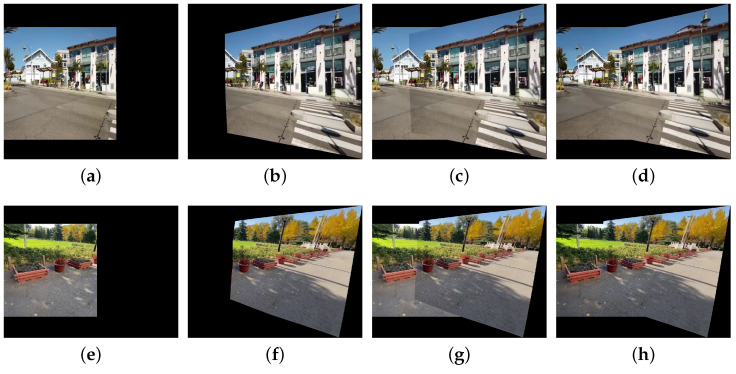
Ablation studies on MRF-based color correction for non-overlapping regions. (**a**,**b**) represent a pair of reference and target images, (**c**) is the corresponding fusion result after performing only the first stage of the correction framework, and (**d**) is the result after applying the complete correction framework. Similarly, (**e**,**f**) represent another pair of reference and target images, while (**g**,**h**) are the respective fusion results obtained by using only the first stage and by using the entire correction framework.

Secondly, we conduct ablation experiments on the second stage of the correction framework—MRF-based color correction for non-overlapping regions—with the results shown in Figure 17. Figure 17a,e represent the target images, while Figure 17b,f are their corresponding reference images. Figure 17c,f show the composition results obtained by using only the first stage—parallax-tolerant color correction for overlapping regions—where noticeable color differences exist between overlapping and non-overlapping regions. In contrast, Figure 17d,h present the results after applying MRF-based color correction for non-overlapping regions, where non-overlapping regions are unified with overlapping regions, resulting in seamless outputs without visible stitching artifacts.

The changes in all the evaluation metrics under different ablation conditions are shown in Table 6. For PSNR and SSIM, which take the reference image as the ground truth, the fewer considerations of parallax in loss functions lead to results where the corrected target image is more similar to the reference image. As a result, PSNR and SSIM achieve their highest values in the absence of dynamic weights and mutation constraints. However, such results do not indicate the best correction performance since the parallax issue is ignored, as shown in Figure 16. Furthermore, since PSNR and SSIM are calculated only for the overlapping regions, the results obtained by using only the first stage and those obtained by using the entire framework are identical in terms of the two evaluation metrics. As for the three no-reference evaluation metrics—BRISQUE, NIQE, and CDCS—since they all reflect the naturalness of the stitching results to some extent, these metrics achieve the best results when using the entire framework and the second-best results when only using the complete first stage. The results from all the above evaluation metrics are consistent with our previous analysis of each metric and the role of different components in each stage, further demonstrating the scientific validity and effectiveness of our PTDCC method.

## 5. Discussion and Conclusions

This paper presents a parallax-tolerant weakly supervised pixel-wise deep color correction method for the image stitching of pinhole camera arrays. First, a parallax-tolerant color correction network based on dynamic loss weights is constructed to compensate for the color differences in overlapping regions. To effectively mitigate the influence of parallax, we construct a convolutional high-dimensional feature extractor to compute the feature vector for each pixel and, based on the differences between feature vectors, we assess the extent of the parallax, thereby dynamically controlling the weight of the loss function. In addition, an inference strategy for correction coefficients is designed based on gradient similarity MRF to harmonize non-overlapping regions with overlapping regions. Furthermore, we innovatively develop an evaluation metric called CDCS that focuses solely on the visibility of stitching artifacts. Comparative experiments conducted on existing evaluation metrics as well as CDCS indicate that our method is capable of handling a wide range of color differences and achieving seamless composition in contrast to existing solutions. The performance of TransUNet, which has been modified accordingly under our correction framework, is second only to our PTDCC. However, its runtime is significantly longer. This also demonstrates the effectiveness of our proposed parallax-tolerant deep color correction framework and the optimal suitability of our U-correction module for this task.

In future work, we aim to develop a proprietary image registration and alignment framework to reduce reliance on existing methods, thereby enabling a fully end-to-end synthesis of natural panoramas. Furthermore, we will focus on transferring this color correction framework to fisheye cameras, allowing our color correction algorithm to be applicable to a broader range of imaging devices and shooting scenarios.

## Figures and Tables

**Table 1:** The parameters of each convolutional block.

	In_Channels	Out_Channels	Kernel_Size	Padding	Dilation
down-sampling block 0	7	16	3	1	1
down-sampling block 1	16	32	3	1	2
down-sampling block 2	32	64	3	1	3
down-sampling block 3	64	128	3	1	4
down-sampling block 4	128	256	3	1	5
up-sampling block 4	256	128	3	1	4
up-sampling block 3	128	64	3	1	3
up-sampling block 2	64	32	3	1	2
up-sampling block 1	32	16	3	1	1
up-sampling block 0	16	3	1	0	1

**Table 2:** Quantitative comparison of overlapping regions’ similarity. The best is marked in red.

	PSNR ↑				SSIM ↑			
	Easy	Moderate	Hard	Average	Easy	Moderate	Hard	Average
UDIS++	20.93	20.14	17.80	20.36	0.774	0.689	0.661	0.727
GLC	25.13	20.72	19.19	22.69	0.793	0.691	0.664	0.737
GCC	23.91	19.90	16.23	21.55	0.787	0.683	0.652	0.730
HHM	25.47	20.92	19.27	22.95	0.793	0.688	0.660	0.735
TransUNet	26.84	22.06	20.53	24.20	0.813	0.711	0.691	0.757
Ours	**29.45**	**26.54**	**44.18**	**29.01**	**0.891**	**0.868**	**0.964**	**0.885**

**Table 3:** Quantitative comparison of composition results’ naturalness. The best is marked in red.

	BRISQUE ↓				NIQE ↓			
	Easy	Moderate	Hard	Average	Easy	Moderate	Hard	Average
UDIS++	38.40	32.92	44.79	36.23	4.20	3.42	4.83	3.87
GLC	37.59	32.57	43.25	35.59	4.19	3.39	4.72	3.85
GCC	37.13	31.88	44.00	35.09	4.10	**3.34**	4.63	3.78
HHM	38.96	33.89	43.70	36.88	4.03	3.36	**4.39**	3.74
TransUNet	37.56	31.26	42.24	38.90	4.15	3.37	4.61	3.81
Ours	**36.01**	**28.72**	**36.57**	**32.63**	**3.88**	3.35	4.45	**3.67**

**Table 4:** Quantitative comparison of CDCS. The best is marked in red.

	CDCS ↓			
	Easy	Moderate	Hard	Average
UDIS++	12.64	12.52	11.48	12.51
GLC	10.73	11.66	9.09	11.06
GCC	11.49	12.00	8.68	11.55
HHM	10.58	11.37	8.87	10.85
TransUNet	10.42	11.14	8.55	10.64
Ours	**10.33**	**10.98**	**7.60**	**10.46**

**Table 5:** Runtime comparison.

	GLC	GCC	HHM	TransUNet	Ours
Runtime	0.218	0.118	0.676	2.453	1.191

**Table 6:** Evaluation metrics of the ablation experiments. The best is marked in red.

	PSNR ↑	SSIM ↑	BRISQUE ↓	NIQE ↓	CDCS ↓
w/o *w* and $Loss_{\gamma }$	**37.35**	**0.985**	33.89	3.75	10.63
w/o *w*	33.78	0.966	34.30	3.71	10.57
w/o $Loss_{\gamma }$	32.66	0.968	33.49	3.71	10.70
w/o the second stage	29.01	0.885	32.99	3.69	10.53
Total framework	29.01	0.885	**32.63**	**3.67**	**10.46**

## Data Availability

The codes used in this research are available at https://github.com/qizhuzhuang/PTDCC, (accessed on 22 January 2025).

## References

[1] Zhu Z., Wei Y., Lu R., Xu C., Le X., Zheng B., Yan C., Xu F. (2024). Indoor Scene Reconstruction using a Rotating Device and Multiple RGB-D Cameras. IEEE Trans. Instrum. Meas..

[2] Li C., Cai C. (2023). A calibration and real-time object matching method for heterogeneous multi-camera system. IEEE Trans. Instrum. Meas..

[3] Fedorov I., Thörnberg B., Alqaysi H., Lawal N., O’Nils M. (2020). A two-layer 3-D reconstruction method and calibration for multicamera-based volumetric positioning and characterization. IEEE Trans. Instrum. Meas..

[4] Yan N., Mei Y., Xu L., Yu H., Sun B., Wang Z., Chen Y. (2023). Deep learning on image stitching with multi-viewpoint images: A survey. Neural Process. Lett..

[5] Fu M., Liang H., Zhu C., Dong Z., Sun R., Yue Y., Yang Y. (2023). Image stitching techniques applied to plane or 3-D models: A review. IEEE Sensors J..

[6] Hsu W.Y., Tsai W.H. (2023). Color Constancy and Color Consistency Using Dynamic Gamut Adjustment. IEEE Trans. Instrum. Meas..

[7] Duplaquet M.L. (1998). Building large image mosaics with invisible seam lines. Proceedings of the Visual Information Processing VII.

[8] Kwatra V., Schödl A., Essa I., Turk G., Bobick A. (2003). Graphcut textures: Image and video synthesis using graph cuts. ACM Trans. Graph. (TOG).

[9] Nie L., Lin C., Liao K., Liu S., Zhao Y. Parallax-tolerant unsupervised deep image stitching. Proceedings of the IEEE/CVF International Conference on Computer Vision.

[10] Cheng S., Yang F., Chen Z., Yuan N., Tao W. (2023). Deep Seam Prediction for Image Stitching Based on Selection Consistency Loss. arXiv.

[11] Shen J., Qian F., Chen X. (2020). Multi-camera panoramic stitching with real-time chromatic aberration correction. J. Phys. Conf. Ser..

[12] Yang S., Wu J., Wang H. (2023). Virtual exhibition image stitching fusion method based on light compensation and improved optimal stitching lines. Proceedings of the 2023 3rd International Conference on Consumer Electronics and Computer Engineering (ICCECE).

[13] Zhang C., Wang D., Sun H. (2023). Image Stitching Based on Color Difference and KAZE with a Fast Guided Filter. Sensors.

[14] Lin D., Li Z., Xin G., Xia Y., Li J. (2023). Research on Colour Correction in Image Stitching With Equal Depth of Field. Proceedings of the 2023 2nd International Conference on Artificial Intelligence, Human-Computer Interaction and Robotics (AIHCIR).

[15] Ding C., Ma Z. (2020). Multi-camera color correction via hybrid histogram matching. IEEE Trans. Circuits Syst. Video Technol..

[16] Yang L., Kong Z., Li T., Bai X., Lin Z., Cheng H. (2023). GPU Accelerated Color Correction and Frame Warping for Real-time Video Stitching. arXiv.

[17] Jeong H., Yoon B., Jeong H., Choi K.S. (2021). Multi-view image color correction using 3D point set registration. Proceedings of the 2021 IEEE International Conference on Image Processing (ICIP).

[18] Yu X., Tang X. (2023). Research on Color Correction Processing of Multi-Hyperspectral Remote Sensing Images Based on FCM Algorithm and Wallis Filtering. IEEE Access.

[19] Liu S., Chai Q. (2018). Shape-optimizing and illumination-smoothing image stitching. IEEE Trans. Multimed..

[20] Liu P., Niu Y., Chen J., Shi Y. (2019). Color Correction for Stereoscopic Images Based on Gradient Preservation. Proceedings of the Intelligent Computing: Proceedings of the 2019 Computing Conference, Volume 1.

[21] Wang C., Gao Z., Lu Q. (2020). Parallax-based color correction in image stitching. Proceedings of the 2020 IEEE 5th International Conference on Image, Vision and Computing (ICIVC).

[22] Lo I.C., Shih K.T., Chen H.H. (2021). Efficient and accurate stitching for 360° dual-fisheye images and videos. IEEE Trans. Image Process..

[23] Song D.Y., Lee G., Lee H., Um G.M., Cho D. (2022). Weakly-supervised stitching network for real-world panoramic image generation. Proceedings of the European Conference on Computer Vision.

[24] Zheng C., Xu Y., Chen W., Wu S., Li Z. (2023). Physics-Driven Deep Panoramic Imaging for High Dynamic Range Scenes. Proceedings of the IECON 2023—49th Annual Conference of the IEEE Industrial Electronics Society.

[25] Ronneberger O., Fischer P., Brox T. (2015). U-Net: Convolutional networks for biomedical image segmentation. Proceedings of the Medical Image Computing and Computer-Assisted Intervention—MICCAI 2015: 18th International Conference.

[26] Geman S., Geman D. (1984). Stochastic relaxation, Gibbs distributions, and the Bayesian restoration of images. IEEE Trans. Pattern Anal. Mach. Intell..

[27] Myronenko A., Song X. (2010). Point set registration: Coherent point drift. IEEE Trans. Pattern Anal. Mach. Intell..

[28] Lowe D.G. (2004). Distinctive image features from scale-invariant keypoints. Int. J. Comput. Vis..

[29] Ma J., Zhao J., Tian J., Yuille A.L., Tu Z. (2014). Robust point matching via vector field consensus. IEEE Trans. Image Process..

[30] Guo C., Li C., Guo J., Loy C.C., Hou J., Kwong S., Cong R. Zero-reference deep curve estimation for low-light image enhancement. Proceedings of the IEEE/CVF Conference on Computer Vision and Pattern Recognition.

[31] Xu Y., Li Z., Chen W., Wen C. (2022). Novel intensity mapping functions: Weighted histogram averaging. Proceedings of the 2022 IEEE 17th Conference on Industrial Electronics and Applications (ICIEA).

[32] Zhang J., Wang C., Liu S., Jia L., Ye N., Wang J., Zhou J., Sun J. (2020). Content-aware unsupervised deep homography estimation. Proceedings of the Computer Vision–ECCV 2020: 16th European Conference.

[33] Nair V., Hinton G.E. Rectified linear units improve restricted boltzmann machines. Proceedings of the 27th International Conference on Machine Learning (ICML-10).

[34] Nie L., Lin C., Liao K., Liu S., Zhao Y. (2021). Unsupervised deep image stitching: Reconstructing stitched features to images. IEEE Trans. Image Process..

[35] Kingma D.P., Ba J. (2014). Adam: A method for stochastic optimization. arXiv.

[36] Chen J., Lu Y., Yu Q., Luo X., Adeli E., Wang Y., Lu L., Yuille A.L., Zhou Y. (2021). Transunet: Transformers make strong encoders for medical image segmentation. arXiv.

[37] Deng J., Dong W., Socher R., Li L.J., Li K., Fei-Fei L. (2009). Imagenet: A large-scale hierarchical image database. Proceedings of the 2009 IEEE Conference on Computer Vision and Pattern Recognition.

[38] Mittal A., Moorthy A.K., Bovik A.C. (2012). No-reference image quality assessment in the spatial domain. IEEE Trans. Image Process..

[39] Mittal A., Soundararajan R., Bovik A.C. (2012). Making a “completely blind” image quality analyzer. IEEE Signal Process. Lett..

